# The value of MRI for downgrading of breast suspicious lesions detected on ultrasound

**DOI:** 10.1186/s12880-023-01021-6

**Published:** 2023-06-05

**Authors:** Zongyu Xie, Wenjie Xu, Hongxia Zhang, Li Li, Yongyu An, Guoqun Mao

**Affiliations:** 1 Department of Radiology, The First Affiliated Hospital of Bengbu Medical College, Bengbu, 233004 Anhui Province China; 2 The Second Clinical Medical College of Zhejiang, Chinese Medical University, Hangzhou, 310053 China; 3 Department of Radiology, Tongde Hospital of Zhejiang Province, Hangzhou, 310012 Zhejiang Province China; 4 Department of Ultrasonography, Tongde Hospital of Zhejiang Province, Hangzhou, 310012 Zhejiang Province China; 5 Department of Radiology, The First Affiliated Hospital of Zhejiang Chinese Medical University (Zhejiang Provincial Hospital of Chinese Medicine), 310006 Hangzhou, China

**Keywords:** Breast, BI-RADS, Magnetic Resonance Imaging, Ultrasound

## Abstract

**Background:**

Most of suspicious lesions classified as breast imaging reporting and data system (BI-RADS) 4A and 4B categories on ultrasound (US) were benign, resulting in unnecessary biopsies. MRI has a high sensitivity to detect breast cancer and high negative predictive value (NPV) to exclude malignancy. The purpose of this study was to investigate the value of breast MRI for downgrading of suspicious lesions with BI-RADS 4A and 4B categories on US.

**Methods:**

Patients who underwent breast MRI for suspicious lesions classified as 4A and 4B categories were included in this retrospective study. Two radiologists were aware of the details of suspicious lesions detected on US and evaluated MR images. MRI BI-RADS categories were given by consensus on the basis on dynamic contrast-enhanced MRI (DCE-MRI) and diffusion-weighted imaging (DWI). Pathological results and imaging follow-up at least 12 months were used as a reference standard. Sensitivity, specificity, positive predictive value (PPV), NPV and their 95% confidence interval (CI) were calculated for MRI findings.

**Results:**

One sixty seven patients with 186 lesions (US 4A category: 145, US 4B category: 41) consisted of the study cohort. The malignancy rate was 34.9% (65/186). On MRI, all malignancies showed true-positive results and 92.6% (112/121) benign lesions were correctly diagnosed. MRI increased PPV from 34.9% (65/186) to 87.8% (65/74) and reduced the false-positive biopsies by 92.6% (112/121). The sensitivity, specificity, PPV and NPV of MRI were 100% (95% CI: 94.5%-100%), 92.6% (95% CI: 86.3%-96.5%), 87.8% (95% CI: 78.2%-94.3%) and 100% (95% CI: 96.8%-100%), respectively. 2.2% (4/186) of suspicious lesions were additionally detected on MRI, 75% (3/4) of which were malignant.

**Conclusion:**

MRI could downgrade suspicious lesions classified as BI-RADS 4A and 4B categories on US and avoided unnecessary benign biopsies without missing malignancy. Additional suspicious lesions detected on MRI needed further work-up.

## Background

According to global cancer statistics in 2018, breast cancer is the most common cancer in women and is the leading cause of death associated with cancer [1]. However, mortality rate of breast cancer has reduced over decades, one of the reasons is detection of breast cancer at an early stage by imaging tools [2]. Ultrasound (US) is the commonly used imaging technique for detection of breast cancer due to its convenience and cost-effectiveness [3, 4].

According to breast imaging reporting and data system (BI-RADS) lexicon, biopsy is recommended for suspicious lesions classified as BI-RADS 4 category. However, positive predictive value (PPV) of BI-RADS 4A and 4B categories on US is relatively low [5–7], implying that numerous benign biopsies are unnecessary. Although US-guided biopsy has advantages of convenience, low cost and high accuracy, it is challenging to perform biopsy in some situations, for instance, lesions are multiple, patients have contraindication for biopsy. In such clinical settings, additional noninvasive diagnostic imaging test with a high sensitivity would be most welcome.

MRI is a noninvasive modality in breast imaging for plenty of indications, for example, screening of high-risk patients, preoperative staging for newly diagnosed breast cancer [8]. Compared with mammography and US, MRI has a high sensitivity of 90%-100% for breast cancer detection [8, 9]. Moreover, negative findings on MRI are reliable to exclude malignancy due to its high negative predictive value (NPV) [9, 10]. Several studies have found MRI has great values in evaluation of inconclusive findings on mammography [11–14]. However, few studies investigated the value of MRI exclusively on suspicious findings on US [15].

Therefore, the purpose of our study is to assess the value of MRI for suspicious lesions classified as US BI-RADS 4A and 4B categories.

## Methods

### Patients

One thousand three hundred four patients who underwent breast MRI examination during March 2014 to November 2019 were included in the study. Inclusion criteria of our study were as follows: (1) clinical indication of breast MRI as a problem-solving tool due to suspicious findings with BI-RADS 4A or 4B category on US; (2) breast MRI prior to surgery or biopsy; (3) presence of either pathological results or imaging follow-up at least 12 months. 209 patients underwent breast MRI for equivocal findings classified as 4A and 4B categories on US. Among them, 40 patients were excluded for absence of pathological results and imaging follow-up at least 12 months. Finally, 167 patients with 186 lesions consisted of the study cohort. The study complied with the Declaration of Helsinki guidelines and declaration. The study was approved by the Ethic Committee of Tongde Hospital of Zhejiang Province. Written inform consent was waived by the Ethic Committee of Tongde Hospital of Zhejiang Province due to the retrospective nature of the study.

### Image protocol

US examinations were performed by experienced sonographers using 5–15 MHz linear-array broadband transducers (Mylab 90, Esaote, Italy; Logiq 9, GE Healthcare, USA; Epiq 5, Philips Healthcare, Netherlands). All patients underwent MRI examinations by a 3.0 T scanner (Verio, Siemens Healthcare, Germany) with a dedicated 8-channal breast coil. MR Images were acquired in axial views. MRI scan protocol consisted of T1-weighted 3D-FLASH sequence (TR 5.9 ms, TE 2.2 ms), T2-weighted turbo inversion recovery magnitude sequence (TR 4000 ms, TE 70 ms), diffusion-weighted imaging (DWI) (TR 6500 ms, TE 85 ms, b = 50, 400, 800 s/mm^2^), and dynamic contrast-enhanced sequences, which included one pre-contrast scan followed by five post-contrast series using fat-suppressed T1-weighted gradient echo sequence (TR 4.6 ms, TE 1.6 ms). Contrast agent (Gd-DTPA, Beilu Pharmaceutical CO., Beijing, China) was injected intravenously at a rate of 2.5 ml/s with a dose of 0.1 mmol/kg, followed by 20 ml saline flush.

### Image interpretation

An experienced sonographers evaluated US images and classified lesions into mass and non-mass, the latter included ductal hypoechoic area with or without calcification, focal nonductal hypoechoic area and architectural distortion [16]. Two radiologists evaluated MR images by consensus. The readers were aware of details of suspicious lesions (size, location, imaging features) detected on US but were blind to pathological results and clinical diagnosis. Additionally, apparent diffusion coefficient (ADC) maps that were automatically generated by the scanner software were accessible to the readers. MRI BI-RADS 4–5 categories were considered as malignant, while 1–3 categories were considered as benign. Furthermore, additional findings without correlation on US that classified as BI-RADS 3–5 categories were recorded.

### Reference standard

Histopathology and imaging follow-up at least 12 months were used as standard of reference. Pathological results were established either by biopsy or surgical resection. Lesions with stability were considered as benign after imaging follow-up (US alone or supplemented with mammography).

### Statistical analysis

SPSS (version 20, IBM Corp, Chicago, USA) and MedCalc (version 15.6.1, MedCalc Software bvba, Ostend, Belgium) was used for statistical analysis. All calculations were performed on a per-lesion basis. Continuous variables were expressed as mean ± standard deviation and compared by Student *t* test or Mann–Whitney *U* test basing on its distribution (normal or non-normal). Categorical variables were expressed as proportions (%) and compared by Chi-square test or Fisher's exact test. Two-tailed *p* value < 0.05 was considered statistically significant. Sensitivity, specificity, PPV, NPV and their 95% confidence interval (95% CI) of MRI were calculated.

## Results

### Study cohort

Among 167 patients, 10.8% (18/167) have two or three lesions detected on US. The mean age of the study population was 50.8 ± 11.2 years (range, 29–85 years). There were 145 lesions classified as 4A category and 41 lesions as 4B category on US. The average size of lesions was 17.4 ± 15.5 mm (range, 5–150 mm). Characteristics of the study population are listed in Table 1.

**Table 1:** Characteristics of the study population

	Benign (*n* = 121)	Malignant (*n* = 65)
Lesion size (mm)	14.1 ± 15.3	23.7 ± 13.8
Mean age	49.6 ± 10.8	52.9 ± 11.6
Menopausal status^a^		
premenopausal	75	32
postmenopausal	46	33
Breast density^a^		
non-dense	42	29
dense	79	36
Background parenchymal enhancement^a^		
minimal to mild	85	51
moderate to marked	36	14

^a^The numbers of menopausal status, background parenchymal enhancement and breast density are calculated on the basis of the number of lesion, other than number of patients, as several patients had multiple lesions

Pathological results are summarized in Table 2. Two atypical ductal hyperplasia were proven by surgical resection and were grouped into the benign. Of 186 lesions, 13 were proven by biopsy (8 benign and 5 malignant lesions) and 150 were proven by surgical resection (90 benign and 60 malignant lesions). The rest 23 lesions classified as 4A category showed negative MRI findings and and considered benign by imaging follow-up (mean time, 21.6 months; range, 12–42 months). Of 23 lesions, 12 were assigned as MRI BI-RADS 1 cateogy, 6 as MRI BI-RADS 2 cateogy and 5 as MRI BI-RADS 3 cateogy.The malignancy rate in 4A and 4B category was 24.1% (35/145) and 73.2% (30/41), respectively. For 65 malignant lesions, invasive ductal carcinoma was the most common type both in BI-RADS 4A (20/35, 57.1%) and 4B category (22/30, 73.3%). For 98 pathology-proven benign lesions, fibrocystic changes were the most common type in 4A category (52/87, 59.8%), whereas fibroadenomas were the most common type in 4B category (6/11, 54.5%).

**Table 2:** Pathological results of breast lesions classified as US BI-RADS 4A and 4B categories

Pathological results	Number of cases
Malignant	65
invasive ductal carcinoma	42 (64.6%)
ductal carcinoma in situ	12 (18.5%)
borderline or malignant phyllodes tumors	5 (7.7%)
other malignant tumors	6 (9.2%)
Benign^a^	98
fibrocystic changes	53 (54.1%)
fibroadenoma	24 (24.5%)
intraductal papilloma	10 (10.2%)
inflammatory disease	6 (6.1%)
atypical ductal hyperplasia	2 (2.0%)
other benign lesions	3 (3.1%)

Numbers in parenthesis were percentages

^a^23 benign lesions confirmed by imaging follow-up were not included. *BI-RADS* breast imaging reporting and data system

### US features of suspicious lesions with BI-RADS 4A-4B category

There were 155 masses, 31 non-mass lesions (9 ductal hypoechoic area, 21 focal non-ductal hypoechoic area, 1 architectural distortion) on US. The cancer rate in non-mass lesions (41.9%, 13/31) was higher than in mass (33.5%, 52/155), though the difference did not reach statistical significance (*p* = 0.412).

### Diagnostic performance of MRI for suspicious lesions on US

**Fig. 1:**
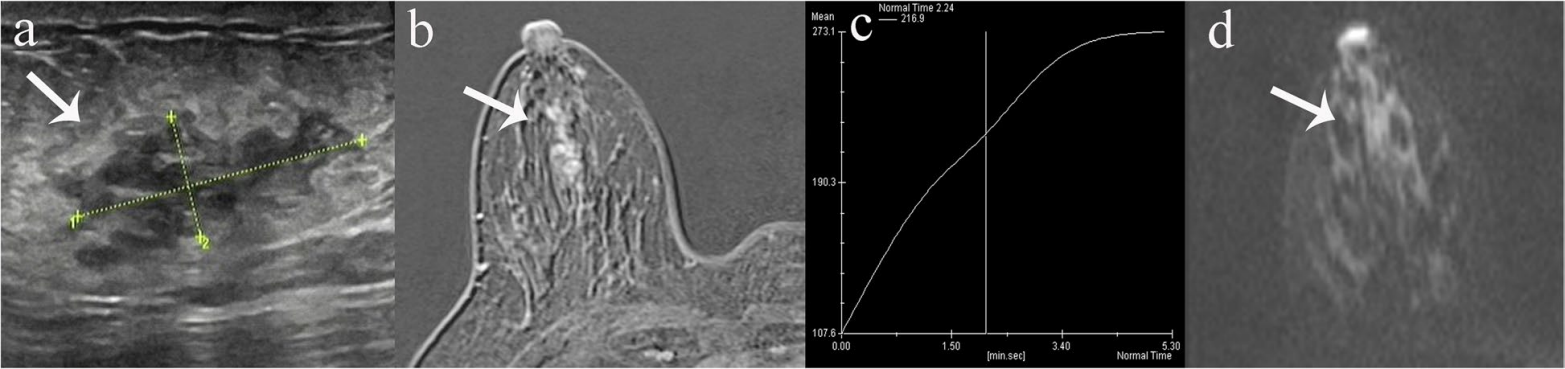
True-negative case of MRI finding for a lesion with US BI-RADS 4A category. **a** Gray-scale US revealed 29 mm non-mass hypoechoic area on the right breast and 4A category was given. **b**, **c** Dynamic contrast-enhanced MRI showed an irregular mass with circumscribed margin and persistent kinetics. **d** The lesion showed slight hyperintensity on DWI, with mean ADC values of 1.51 × 10^–3^ mm^2^/s. BI-RADS 3 category was given on MRI. Pathology revealed fibrocystic changes

**Fig. 2:**
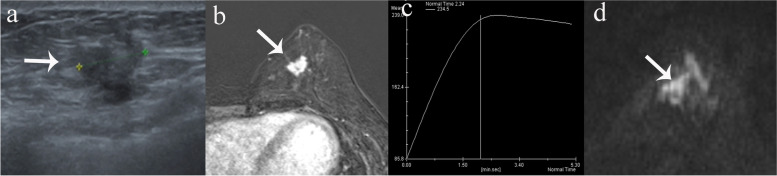
True-positive MRI finding for a lesion with US BI-RADS 4B category. **a** Gray-scale US revealed 14 mm irregular hypoechoic mass with indistinct margin on the left breast and 4B category was given. **b**, **c** Dynamic contrast-enhanced imaging showed an irregular mass with irregular margin and plateau kinetics. **d** The lesions displayed hyperintensity on DWI, with mean ADC values of 0.72 × 10^–3^ mm^2^/s. BI-RADS 5 category was given on MRI. Pathology showed high-grade ductal carcinoma in situ

**Fig. 3:**
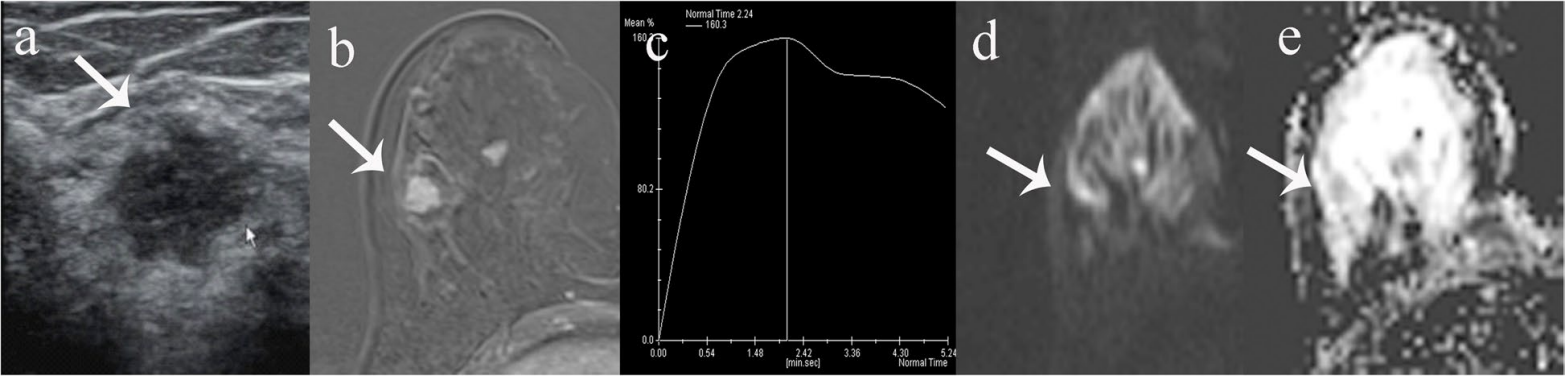
False-positive MRI finding for a lesion with US BI-RADS 4B category. **a** Gray-scale US revealed 13 mm irregular hypoechoic mass with indistinct margin on the right breast and 4B category was given. **b**, **c** Dynamic contrast-enhanced MRI showed an irregular mass with irregular margin and washout kinetics. **d**-**e** The lesion showed hyperintensity on DWI and hypointensity on ADC mapping, with mean ADC values of 1.11 × 10^–3^ mm^2^/s. BI-RADS 4 category was given on MRI. Pathology showed intraductal papilloma

All 65 malignant lesions were correctly diagnosed on MRI (29 MRI BI-RADS 4 category, 36 MRI BI-RADS 5 category). 112 of 121 benign lesions were correctly downgraded to BI-RADS 1–3 categories on MRI. 9 benign lesions were false positive on MRI, including 3 fibrocystic changes, 4 intraductal papillomas, 1 benign phyllodes tumor and 1 atypical ductal hyperlasia. The sensitivity, specificity, PPV and NPV of MRI were 100% (95% CI: 94.5%-100%), 92.6% (95% CI: 86.3%-96.5%), 87.8% (95% CI: 78.2%-94.3%) and 100% (95% CI: 96.8%-100%), respectively. MRI increased PPV from 34.9% (65/186) to 87.8% (65/74) and reduced false-positive biopsies by 92.6% (112 of 121). Diagnostic performances of MRI are listed in Table 3. Examples are listed in Figs. 1, 2 and 3.

**Table 3:** Diagnostic performances of MRI for suspicious lesions classified as US BI-RADS 4A and 4B categories

	Sensitivity (%)	Specificity (%)	PPV (%)	NPV (%)
All lesions	100 (94.5–100)	92.6 (86.3–96.5)	87.8 (78.2–94.3)	100 (96.8–100)
US BI-RADS category				
4A	100 (90–100)	93.6 (87.3–97.4)	83.3 (68.6–93.0)	100 (96.5–100)
4B	100 (88.4–100)	81.8 (48.2–97.7)	93.8 (79.2–99.2)	100 (66.4–100)
Lesion type				
Mass	100 (93.2–100)	92.2 (85.3–96.6)	86.7 (75.4–94.1)	100 (96.2–100)
Non-mass	100 (75.3–100)	94.4 (72.7–99.9)	92.9 (66.1–99.8)	100 (80.5–100)

Numbers in parentheses are 95% confidence intervals. *PPV* positive predictive value, *NPV* negative predictive value

*Abbreviations*: *BI-RADS* Breast imaging reporting and data system, *US* Ultrasound, *DCE-MRI* Dynamic contrast-enhanced magnetic resonance imaging, *PPV* Positive predictive value, *NPV* Negative predictive value, *DWI* Diffusion-weighted imaging, *ADC* Apparent diffusion coefficient, *CI* confidence interval

When stratifying by US BI-RADS category, the sensitivity, specificity, PPV and NPV of MRI findings for lesions with 4A category were 100% (95% CI: 90%-100%), 93.6% (95% CI: 87.3%-97.4%), 83.3% (95% CI: 68.6%-93.0%), 100% (95% CI: 96.5%-100%), respectively, and the corresponding values were 100% (95% CI: 88.4%-100%), 81.8% (95% CI: 48.2%-97.7%), 93.8% (95% CI: 79.2%-99.2%) and 100% (95% CI: 66.4%-100%) for lesions with 4B category.

When stratifying by lesion type on US, diagnostic parameters of MRI findings were as follows: sensitivity of 100% (95% CI: 93.2%-100%), specificity of 92.2% (95% CI: 85.3%-96.6%), PPV of 86.7% (95% CI: 75.4%-94.1%), NPV of 100% (95% CI: 96.2%-100%) in mass compared with sensitivity of 100% (95% CI: 75.3%-100%), specificity of 94.4% (95% CI: 72.7%-99.9%), PPV of 92.9% (95% CI: 66.1%-99.8%), NPV of 100% (95% CI: 80.5%-100%) in non-mass lesions.

### Additional findings on MRI

Four suspicious lesions (2.2%, 4/186) were found additionally on MRI. Patients with additional lesions had synchronous malignancy on the contralateral or ipsilateral breast. Of these lesions, 3 were confirmed malignant by pathology (2 ductal carcinoma in situ and 1 invasive carcinoma) and 1 lesion referred for biopsy was lost to follow-up.

## Discussion

BI-RADS category has been widely used in clinical practice. According to BI-RADS atlas, lesions classified as 4 category warrant biopsy rather than follow-up. However, nearly 90% biopsies prompted by US yielded benign results in a large prospective multicenter ACRIN 6666 trials [17], resulting in increases of medical cost and psychological burden for patients. Although US-guided biopsy is accessible with high accuracy and less harm, it is invasive and may be challenging to perform in some situations. Thus, the purpose of our study is to investigate the value of MRI for suspicious lesions with low-intermediate risk of malignancy detected on US.

Our study indicated MRI was useful in both mass and non-mass lesions detected on US. In our study, no malignancies were misdiagnosed and most of benign lesions could be downgraded. MRI increased PPV from 34.9% (65/186) to 87.8% (65/74) and reduced false-positive biopsies by 92.6% (112/121). Furthermore, 3 of 4 additional suspicious lesions were proven malignant detected on MRI.

There were some controversies on the value of MRI as a problem-solving modality for suspicious clinical and radiological findings. In 2004, a multicenter study by Bluemke et al. [18] involved 821 suspicious mammography or clinical findings revealed a sensitivity of 88.1%, specificity of 67.7%, NPV of 85% of breast MRI, indicating that MRI could not eliminate the need of biopsy due to its moderate specificity. Subsequently, several meta-analyses suggested MRI might not be conclusive as a problem-solving tool for equivocal findings [19–21]. Due to lack of evidence in this setting, European Society of Breast Imaging recommends MRI may not be an alternative to biopsy for equivocal findings at conventional imaging, and can be used only when biopsy can not be performed [8]. However, several recent studies have suggested that MRI can be used as a problem-solving tool for plenty of clinical indication and reduces unnecessary benign biopsy. Strobel et al. [22] obtained a sensitivity of 95.5%, specificity of 92% and NPV of 98.9% of MRI on the suspected mammography and US findings. In a study by Spick et al. [10], negative findings on MRI for lesions with BI-RADS 0 category were reliable to exclude malignancy. Additionally, MRI can also be helpful to decrease unnecessary benign biopsy for suspicious calcification and architectural distortion on mammography [11, 12]. Our study provided additional empirical data and supported the aforementioned results. However, in a retrospective study by Sarica et al. [15], the results suggested MRI might not be effective in the assessment of BI-RADS 4 lesions on US, in discordance with ours. In that study, 5 of 79 benign MRI findings were false negative and 60 of 110 MRI suspicious findings were false positive for US BI-RADS 4 lesions, resulting in a low-moderate specificity of 56.7%, NPV of 46.4%. The reasons for the discrepancy between Sarica's study and ours may be as follows: MR imaging interpretations were evaluated solely on the basis of morphological criteria and kinetics in the study of Sarica et al. [15], and DWI was not incorporated into the evaluation. However, there was some overlap on morphology and kinetics between breast benign and malignant lesions, resulting in a relatively low specificity [23]. Several studies have suggested DWI can be used as a valuable adjunct to improve diagnostic accuracy and alleviate false-positive findings [23–25]. In our study, DWI combined morphological features and kinetics was used to assess breast lesions. Therefore, our study may be more convincing as DWI is widely used in imaging evaluation in clinical practice.

One concern for breast MRI was additionally detected lesions due to its high sensitivity. These additional lesions needed further work-up and potentially limited the usage of diagnostic breast MRI. Previous studies showed detection rate of additional suspicious lesions on MRI was low, but a considerable proportion of these lesions were malignant. Strobel et al. [22] reported 8 of 340 patients (2.4%) had incidental findings, and 3 of 8 (37.5%) were small invasive breast cancer. Spick et al. [26] found that 5.3% (16/302) patients had incidental MRI findings, and 37.5% (6/16) were malignant. In our study, the rate of incidental findings on MRI was 2.2% (4/186), 75% (3/4) of which were malignant. These suspicious additional lesions may have a vital impact on treatment plan, especially for patients with existed breast cancer. In any case, further evaluation is required for the suspicious additional MRI finding. A second-look or MRI-directed US is beneficial in this setting [27].

Another concern for breast MRI was false-negative findings. The reported false-negative rate of MRI was from 0%-7.5% in studies [11, 12, 28]. Majority of false-negative findings were ductal carcinoma in situ, which showed absence of enhancement on MRI. Mammography could assist in reducing false-negative cases of ductal carcinoma in situ, which commonly manifested suspicious microcalcification [29, 30]. Moreover, from pathological point of view, these non-enhanced ductal carcinoma in situ may be biologically dormant [31] and are considered potential overdiagnosis and overtreatment [32]. To sum up, the benefits of MRI to solve suspicious findings and avoid substantial unnecessary benign biopsies outweighed its negative impact.

The PPV was 24.1% (95% CI: 17.4%-31.9%) for US BI-RADS 4A category and 73.2% (95% CI: 57.1%-85.8%) for 4B category in our study, higher than that of ACR benchmarks (4A: 2%-10%; 4B: 10%-50%). Previous studies showed inconsistent results of PPV of US BI-RADS 4 category. Zou et al. [33] and He et al.[7] obtained PPV of 23.7% (52/219) and 70.7% (53/75) versus 13.6% (100/733) and 50% (136/272) for BI-RADS 4A and 4B category respectively. On the one hand, the US BI-RADS subcategories are assessed predominantly depending on experience of doctors rather than objective criteria. Some doctors are very cautions and prefer a lower subcategory in order to decrease anxiety and mental burden of patients. [7, 33] On the other hand, plenty of benign findings were excluded in our study, as some clinicians opted for US-guided biopsy instead of further MRI examination. The selection bias may contribute to high PPV of US 4A and 4B categories in our study.

There are several limitations in our study. Firstly, this was a single center study and the sample size was relatively small. Secondly, 23 lesions assigned as US 4A categroy showed negative findings on MRI and thus biopsy was not performed. These lesions were considered benign by imaging follow-up with mean time of 21.6 months, which may lead to overestimating sensitivity and NPV of MRI. However, among theses 23 lesions, only 5 lesions were classified as MRI BI-RADS 3 category and the rest were classified as MRI BI-RADS 1–2 categories. In view of the low malignancy likelihood of MRI BI-RADS 3 category [34] and approximate 100% of NPV of MRI to exclude malignancy [9, 10, 22], unknown pathology of these 23 lesions may have minor impact on the results. Finally, we did not assess inter-observer variability in imaging analysis among radiologists. In our study, two radiologists evaluated the imaging separately and reached a consensus for disagreement, this double-reading pattern is consistent with daily work practice.

In conclusion, our study indicates MRI yielded high diagnostic performance in suspicious lesions classified as US BI-RADS 4A and 4B category. Breast MRI could be used as a problem-solving tool in such clinical settings to reduce unnecessary benign biopsies without missing malignancy. Furthermore, additional suspicious lesions detected on MRI needed further work-up.

## Data Availability

The data that support the findings of this study are available from the corresponding author upon reasonable request.
